# Vitrification of Mouse MII Oocyte Decreases the Mitochondrial DNA Copy Number, TFAM Gene Expression and Mitochondrial Enzyme Activity

**Published:** 2017

**Authors:** Mahboobeh Amoushahi, Mojdeh Salehnia, Seyed Javad Mowla

**Affiliations:** 1- Department of Anatomy, Tarbiat Modares University, Tehran, Iran; 2- Department of Biotechnology, Faculty of Biological Sciences, Tarbiat Modares University, Tehran, Iran

**Keywords:** Cytochrome c oxidase, mtDNA copy number, Reactive oxygen species, Succinate dehydrogenase

## Abstract

**Background:**

The objective of this study was determination of the changes in the reactive oxygen species (ROS) level, mitochondrial DNA (mtDNA) copy number and enzyme activity and transcription factor A (TFAM) gene expression in oocytes after vitrification.

**Methods:**

The oocytes at metaphase II (MII) stage (n=320) were collected from super-ovulated adult female mice (n=40). These oocytes were divided into vitrified and non-vitrified groups (n=160 in each group). After vitrification of oocytes, ROS level, mtDNA copy number; TFAM gene expression and mitochondrial enzymes activity (cytochrome C oxidase and succinate dehydrogenase) were assessed and compared with non-vitrified group. Visualization of the mitochondria was done using Mitotracker green staining under confocal microscope. Data were compared by independent T-test. Values of p<0.05 were considered as statistically significant.

**Results:**

The survival rate of oocytes after vitrification and warming was 96.05%. The intensity of cytochrome C oxidase activity, mtDNA copy number and TFAM gene expression in non-vitrified oocytes were significantly lower and the level of ROS was higher in vitrified oocytes in comparison with non-vitrified group (p<0.05). But the intensity of succinate dehydrogenase activity was not significantly different between the two groups. The pattern of mitochondrial distribution in two groups of study was similar but the intensity of Mitotracker green in non-vitrified oocytes was significantly higher than vitrified oocytes (p<0.05).

**Conclusion:**

This study showed that vitrification of mouse MII oocytes reduced the mtDNA copy number and mitochondrial cytochrome C oxidase activity by increasing ROS level, thus the subsequent embryo development may be affected.

## Introduction

Oocyte cryopreservation is a suitable method in assisted reproductive technologies for fertility preservation (1, 2). Slow freezing and vitrification have been employed as oocyte cryopreservation methods (3, 4). Vitrification is a physical process in high concentrated cryoprotectants without formation of any ice crystals. This method is a good alternative to slow-cooling method (5, 6).

Recently, vitrification methods have been improved using enhancement of cooling rate, decline in the volume of cryoprotectant and different carrier systems (7–9). In spite of the successful results in vitrification techniques, there are studies that have showed various structural, biochemical and molecular alterations after vitrification process which result in loss of oocyte quality (10–13).

Cytoplasmic maturation requires high level of energy which is provided by mitochondria (14). This organelle has vital role in production of energy for oocyte; therefore, it is an indicator of cytoplasmic maturation (15, 16). Mitochondria of oocyte may be damaged without any detectable morphological alterations during cryopreservation process (17).

Mitochondrial genome consists of a circular, double-stranded DNA molecule with 16.6 *kb*. It codes 22 tRNA, 2 rRNA and 13 necessary subunits of the respiratory chain complexes (18, 19). The mtDNA copy number has a link with oocyte quality and plays vital role in production of ATP within the oocyte (20, 21).

ROS is produced by mitochondrial respiratory chain and may alter the mtDNA copy number (19). Alteration in the activity of the enzymes involved in oxidative phosphorylation system (OXPHOS) may be due to decline mtDNA copy number (22, 23). One of the vital enzymes in respiratory chain is cytochrome oxidase (COX) that produces water without ROS production by reaction with oxygen (24). Unlike other complexes, complex II or succinate dehydrogenase (SDH), does not participate in transfer of proton. SDH not only participates in electron transfer in respiratory chain but also has a principal role as an enzyme in crebs cycle, thus this enzyme plays an important role in energy metabolism. Additionally, SDH plays a major role in connecting two pathways of cellular metabolism (25). 13 subunits of complex I, III, IV and V encode by mitochondrial genome but other subunits of these complexes and total subunits of SDH encode by nuclear genome. However, correlation between nuclear and mitochondrial genome is important for mitochondrial function (26). The maintenance and stability of mitochondrial genome is supported by transcription factor A mitochondria (TFAM). Transcription and replication of mitochondrial DNA (mtDNA) play a critical role in mitochondrial biogenesis during development (27).

It was shown that cryopreservation may change the physical and chemical characteristics of oocytes, such as loss of cytoskeletal integrity, mitochondrial depolarization, and increase in level of ROS (28–34).

Recent studies demonstrated that vitrification and warming process may impact on mitochondrial function, structure and distribution of oocytes (35–39). Lei et al. reported that vitrification changed mitochondrial distribution and function in mouse oocytes and had negative effect on fertilization and embryonic development (35). Shi et al. showed changes in mitochondrial organization and high sensitivity of porcine oocytes at MII stage to vitrification process (37). Results of Nazmara et al.’s study revealed that mouse oocyte vitrification did not affect the developmental competence and ATP content but some alterations were seen in mitochondrial distribution of *in vitro* matured oocytes compared with control group (38).

In spite of some reports regarding the adverse effects of oocyte cryopreservation (40–44), there was poor information related to the changes in mtDNA copy number and mitochondrial enzyme activity of oocytes after vitrification. Therefore, the object of this study was determining the alterations in mtDNA copy number, ROS level, mitochondrial enzyme activity, TFAM gene expression and mitochondrial distribution in vitrified oocytes compared with non-vitrified group.

## Methods

### Chemicals:

All reagents were purchased from Sigma Aldrich (Germany) except otherwise indicated.

### Animals:

Adult female (6–10 weeks old; n=40) NMRI mice were cared and used according to the guide for the care and use of laboratory animals of Tarbiat Modares University, Tehran, Iran. The mice were housed under a 12 *hr* light: 12 *hr* dark regimen with a temperature of 23°*C* and relative humidity of 44%. Approval for this study was achieved from the animal research ethical committee of Tarbiat Modares University (Ref No: 52/1637).

### Ovulation induction:

The fresh MII oocytes were collected from adult female mice (n=40) after superovulation by intraperitoneal (IP) injection of 10 *IU* human menopausal gonadotrophin (HMG, Folligon; Intervent, Australia) followed with another injection of 10 *IU* of hCG at 48 *hr* later.

### Experimental design:

The collected MII oocytes (n=320) from adult female mice were randomly divided into vitrified and non-vitrified groups (n= 160 in each group) and in each group subjected to subsequent assessments.

The oocyte level of ROS, TFAM gene expression, mtDNA copy number and mitochondrial enzymes activity in both groups were assessed and compared.

### Vitrification and warming:

Oocytes (n=160 oocyte) were vitrified by cryotop method described by Kuwayama (45). Briefly, the oocytes were placed in equilibration solution, containing 7.5% DMSO, 7.5% ethylene glycol and 20% human serum albumin (HSA) in Ham’s F10 at room temperature for 7 *min*. Then, the oocytes were placed in vitrification solution, containing 15% DMSO, 15% ethylene glycol, 0.5 *mol* sucrose and 20% HSA in Ham’s F10 for 1 *min*. The oocytes were loaded to cryotop and then plunged into liquid nitrogen. For warming, the cryotop was immersed into a solution of 1 *mol* sucrose containing 20% HSA in Ham’s F10 at 37°*C* for 1 *min*. Next, the oocytes were sequentially transferred into a solution of 0.5 *mol* sucrose for 3 *min* as well as a solution of 0.25 *mol* sucrose for 3 *min* and washed with a medium supplemented with 20% HSA. Then, they were incubated for 1 *hr* before any assessments.

### DNA extraction from individual oocyte:

For DNA extraction, a single MII oocyte (n=40 in each group, n=1 per tube) was transferred into 10 *μl* of lysis buffer containing 50 *mM* Tris-Hcl (pH=8.5), 0.1 *mM* EDTA, 0.5% Tween-20 and 200 *μg/ml* proteinase K (Roch, Germany). Next, each tube was incubated at 55°*C* overnight. For inactivation of proteinase K, they were heated to 95°*C* for 10 *min*. For PCR, each sample was directly employed as template DNA.

### Primer design:

The unique regions of mitochondrial genome without duplicate in nuclear genome were detected and designed in specific primers (Table 1) by primer3 (version 4.0) software. These primers were synthesized at Generary Biotech Co. (China).

**Table 1.** Designed primer sequences used for real-time PCR

**Gene**	**Primer pair sequence (5′-3′)**	**Accession numbers**	**PCR product size (*bp*)**
**β-actin**	F: TGTGACGTTGACATCCGTAA R: GCTAGGAGCCAGAGCAGTAA	NM-007393	64
**TFAM**	F: AAGGGAATGGGAAAGGTAGA R: AACAGGACATGGAAAGCAGAT	NM-011045	76
**MT**	F: CCAATACGCCCTATAACAAC R: GCTAGTGTGAGTGATAGGGTAG	NM-013523.3	79

### Preparation of standard dilutions:

A 68 *bp* unique sequence of mtDNA was amplified using mitochondrial forward and mitochondrial reverse primers. PCR products were extracted from agarose gel using GeneAll Expin^TM^ Combo GP kit (GeneAll Biotechnology, Korea), according to the manufacturer’s protocol. Afterward, this product was cloned into the vector pTZ57R/T (Thermoscientificbio, USA), purified and sequenced. Linearization and cleanup of recombinant plasmid was done by GeneAll kit. Concentration of this plasmid was detected and diluted to 3×10^5^ copies/5 *μl* by spectrophotometry. Preparation of five serial dilutions of purified recombinant was performed. These standard serial dilutions were stored at 4°*C* for mtDNA copy number analysis.

### Quantification of mtDNA copy number:

Real time PCR was performed in each single MII oocytes (n=40 in each group, n=1 per tube) for quantification of mtDNA copy number.

Duplicates of extracted mtDNA from each oocyte were used as 2 wells. Five points of triplicate serial standard without template control were employed in all real-time analysis. The cycling programs consisted of an initial denaturation step of 95°*C* for 10 *min*, followed by 40 cycles of 95°*C* for 15 seconds, 60°*C* for 30 *s* and 72°*C* for 30 *s*. Duplicate wells of each oocyte were summed for determination of mtDNA copy number (R^2^=0.99).

### RNA extraction for TFAM:

Total RNA was extracted from vitrified and non-vitrified MII oocytes (30 oocytes in each group; 10 oocytes for each replicate of experiments) using RNeasy Mini Kit (Qiagen, Germany). RNA samples were stored at −80°*C*. According to the manufacturer’s instructions, the cDNA was synthesized using the cDNA kit (Thermo Scientific, UK). The cDNA was synthesized by oligodT and reverse transcriptase at 42°*C* for 60 *min* then, stored at −20°*C*.

### Real-time RT-PCR:

Primer pairs for TFAM gene expression were designed by GenBank (http://www.ncbi.nlm.nih.gov) and Allele ID software and are presented in table 1. β-actin was utilized as a housekeeping gene.

Applied Biosystem real time thermal cycler was utilized according to QuantiTect SYBR Green RT-PCR kit (Applied Biosystems, UK). Amplification of reference and target genes was performed in the same run, for each sample. Programs of real time RT-PCR protocol consisted the holding step at 95°*C* for 5 *min*, cycling step at 95°*C* for 15 *s*, 58°*C* for 30 *s*, and 72°*C* for 15 *s*, which was followed by a melt curve step at 95°*C* for 15 *s*, 60°*C* for 1 *min*, and 95°*C* for 15 *s*. Determining relative quantitation for target genes was performed by Pfaffl method. All experiments of real time RT-PCR were replicated three times.

### ROS assay:

ROS level of MII oocytes (60 oocytes for each group in 3 repeats) from two groups was set based on Abdi et al.’s method (46). Briefly, the 20 oocytes were pooled in each experiment and washed three times with PBS; afterward, they were incubated in 40 *mmol/l* of Tris–HCl buffer (pH=7.0) containing 5 *mmol/l* 2^′^, 7′ dichlorodi-hydrofluorescein diacetate (Merck) at 37°*C* for 30 *min*. They were washed with PBS, sonicated at 50W for 2 *min*, centrifuged at 4°*C* and 10,000 *g* for 20 *min*, and the supernatant was monitored using a spectrofluorometer at 488 *nm* excitation and at 525 *nm* emissions.

### Cytochrome C oxidase activity:

Activity of cytochrome C oxidase in MII oocytes (n=10 in each group) was determined by the method described by Burstone et al. (47). Briefly, the MII oocytes were placed in a solution of 15 *mg* N-Phenyl-p-phenylene diamine (p-aminodiphenylamine), 15 *mg* naphthol AS-LG 0.1 *ml* ethanol 100%, 50 *ml* tris-HCl buffer (pH=7.2–7.4) and BSA (5 *mg/ml*) after washing in phosphate-buffered saline supplemented with 5% BSA and then, incubated at 37°*C* for 60 *min*. The reaction was inhibited by 1.10 *mM* potassium cyanide.

### Succinate dehydrogenase activity:

The activity of succinate dehydrogenase in MII oocytes (n=10 in each group) was detected by the method described by of Vivarelli (48).

Briefly, the oocytes were washed in phosphate-buffered saline supplemented with bovine serum albumin. Then, they were transferred in one drop (0.8 *ml*) of a solution containing 12.5 *mM*-tris-HCl (pH=7.4); 1.25 *mg* BSA/*ml*; 1.25 *mM*-CaCl2; 1 *mM*-NaCN; 62.5 *mM*-disodium succinate (Boehringer). Immediately the MII oocytes were loaded on cryotop with small volume of the same solution and were frozen. After thawing the oocytes at 37°*C*, 0.1 *ml* of nitro blue tetrazolium (B.D.H.) was added and incubated at 37°*C* for 15 *min*. For controls, MII oocytes were incubated in the same solution containing 90 *mM*-NaCl instead substrate.

### Quantization of enzymes activity:

For determination of cytochrome c oxidase and succinate dehydrogenase activity, stained MII oocytes were transferred in the center of glass slide. These slides were observed under a light microscope. The intensity of the enzyme activity was determined by the amount of deposited reaction products. The light microscopic images of individual oocytes (n=5 in each groups) were taken at 400 magnification and entered into Image J software (National Institutes of Health, Bethesda, USA). The intensity of reaction in each oocyte was measured and compared by this software.

Activity of cytochrome C oxidase in MII oocytes (n=10 in each group) were determined with the method described by Burstone et al (47). Briefly, the MII oocytes were washed in phosphate-buffered saline supplemented with 5% BSA, then they were incubated in a solution containing 15 *mg* N-Phenyl-p-phenylene diamine (p-aminodiphenylamine), 15 *mg* naphthol AS-LG, 0.1 *ml* ethanol 100%, 50 *ml* tris-HCl buffer (pH=7.2–7.4) and 5 *mg* BSA/*ml* at 37°*C* for 60 *min*. In negative control the reaction was inhibited by 0.1 *mM* potassium cyanide.

### Statistical analysis:

All experiments were repeated at least three times. Values are given as mean±SE. For data evaluation, the SPSS program version 21 software was utilized. The normality of data was tested by the Kolmogorove-Smirnov Test. The data of mtDNA copy number, TFAM gene expression, ROS level, enzyme activity and fluorescent intensity in the vitrified and non-vitrified groups were compared with independent t-test. The p<0.05 was considered as statistically significant.

## Results

### mtDNA copy number of MII oocytes:

The mtDNA copy numbers of non-vitrified and vitrified MII oocytes were 511764.8±124899 and 243704± 28450, respectively (Table 2). This number in vitrified MII oocytes was lower than non-vitrified group (p<0.001).

**Table 2.** The mtDNA copy number, the ratio of TFAM gene expression to β-actin and the ROS level in non-vitrified and vitrified MII oocytes (M±SEM)

**Group**	**mtDNA copy number**	**Relative expression ratio of TFAM to β-actin**	**ROS levels (*μ*M H_2_O_2_)**
**Non-vitrified**	511764.8±124899	0.33±0.08	2.64±0.03
**Vitrified**	243704±28450*	0.04±0.01*	4.39±0.11*

* Significant difference with non-vitrified group (p<0.001)

### Gene expression analysis:

The gene expression ratios of TFAM to housekeeping gene in vitrified and non-vitrified MII oocytes were 0.33±0.08 and 0.04±0.01, respectively (Table 2). The gene expression in non-vitrified MII oocytes was significantly higher than vitrified oocytes (p<0.001).

### ROS level:

The ROS levels in MII oocytes collected from two groups of study were indicated as *μM* H_2_O_2_ (Table 2). The amounts of ROS in non-vitrified and vitrified groups were 2.64±0.03 and 4.39±0.11. These levels in vitrified MII oocytes were higher than non-vitrified group (p<0.001).

### Cytochrome C oxidase activity:

**Figure 1.**
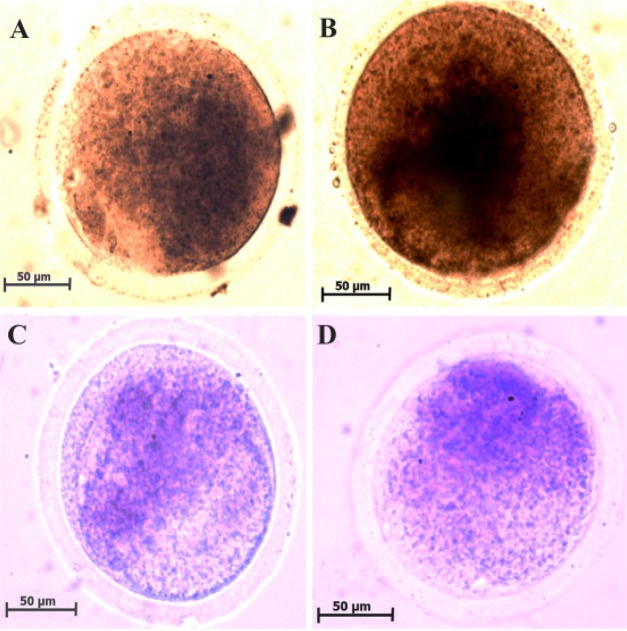
Representative photomicrographs of cytochrome C oxidase (A and B) and succinate dehydrogenase (C and D) reaction in MII oocytes collected from two groups of study. The cytochrome C oxidase activity is shown as brown color in non-vitrified (A) and vitrified (B) MII oocytes. The succinate dehydrogenase activity is shown as purple color in non-vitrified (C) and vitrified (D) MII oocytes

Cytochrome C oxidase reaction is shown bluish brown to brownish black in the micrograph of MII oocytes (Figure 1). The intensities of enzyme activity in non-vitrified and vitrified MII oocytes were 178.13±0.83 and 160.38±3.47, respectively (Table 3). These reactions in non-vitrified MII oocytes were significantly higher than vitrified oocytes (p<0.001).

**Table 3.** The intensity of mitochondrial enzyme activity in non-vitrified and vitrified groups

**Groups**	**Cytochrome c oxidase**	**Succinate dehydrogenase**
**Non-vitrified**	178.13±0.83	222.86±4.97
**Vitrified**	160.38±3.47 *	209.20±6.64

* Significant difference with non-vitrified group (p<0.05)

### Succinate dehydrogenase activity:

Purple formazan deposit was observed in MII oocytes with succinate dehydrogenase reaction (Figure 1). The intensities of succinate dehydrogenase activity in non-vitrified and vitrified MII oocytes were 222.86±4.97 and 209.20±6.64 (Table 3). There was no significant difference between two groups.

### Distribution of mitochondria:

**Figure 2.**
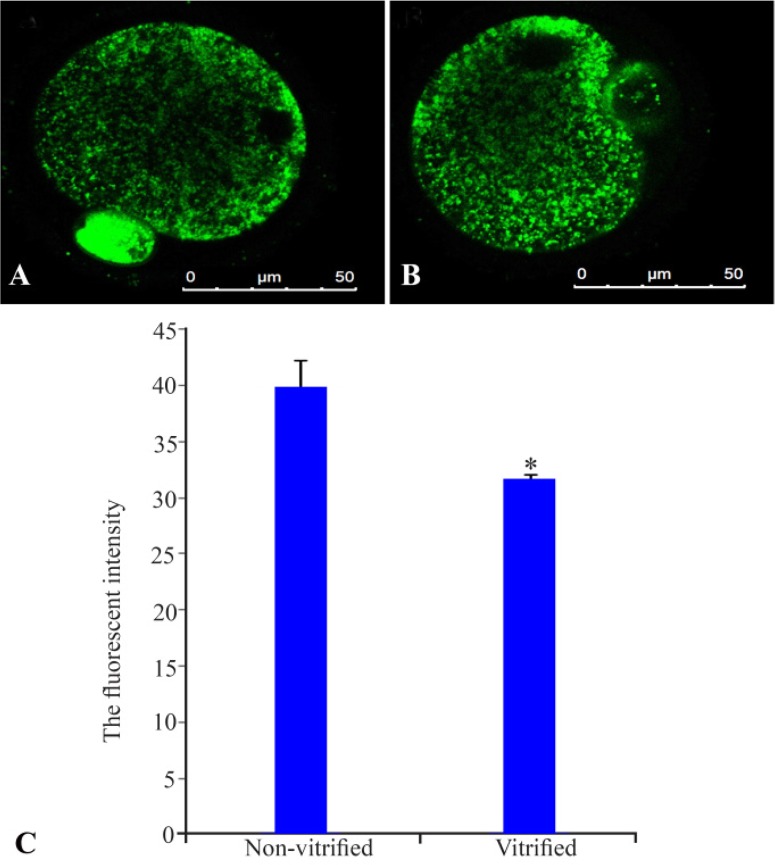
Distribution of mitochondria in non-vitrified (A) and vitrified (B) MII oocytes that were stained by Mitotracker green. The mitochondria are shown as green clusters within the ooplasm. The fluorescence intensity of MII oocytes in two groups of study (C). * Significant differences with non-vitrified group (p<0.05)

The representative micrographs of MII oocytes which stained with Mitotracker green are observed in figures 2A and 2B. Mitochondrial distribution in the cytoplasm of MII oocytes was homogenous, although distribution of mitochondria in cortical region of cytoplasm was more than central region. This pattern in two groups of study was similar. The intensities of florescent in non-vitrified and vitrified MII oocytes were 39.80±1.42 and 31.66±0.22 (Figure 2C). These intensities in non-vitrified MII oocytes were significantly higher than vitrified oocytes (p<0.05).

## Discussion

These results showed that vitrification and warming process may result in mitochondrial deficiency of oocytes. Our result showed that vitrification process leads to increase in ROS level of oocyte. Similarly, Nohales-Córcoles et al. reported that vitrification affected the redox state of human oocytes (50). Gupta et al. observed that vitrification enhanced the ROS activity and reduced the quality of oocytes (51).

Increase in ROS production may lead to impairment in respiratory chain as the main producer of ROS (52–56). Therefore, impaired respiratory chain, in turn, may lead to increase in ROS production and high level of ROS may lead to defect in mtDNA copy number. Results of this study showed that mtDNA copy number in vitrified oocytes was significantly decreased in comparison with non-vitrified oocytes. Decrease in mtDNA copy number may be due to increase in ROS level.

Osmotic forces due to dehydration, cooling, rehydration, and warming may cause defect in mitochondrial structure, function or distribution (17, 30, 33, 57, 58). The mtDNA copy number directly links with quality of oocytes (20, 21, 59, 60).

Some of investigations showed that vitrification methods by various careers and cryoprotectants impacted on mitochondrial structure and function (17, 30, 33, 61, 62). Chen et al. reported that vitrification changed mitochondrial membrane potential of human oocytes (63). Dai et al. observed that vitrification process impaired mitochondrial morphology and function of porcine oocytes (40).

In other part of this study, it was shown that cytochrome oxidase activity in vitrified MII oocytes was lower than non-vitrified group. This result is in parallel with decrease in mtDNA copy number and increase in ROS production. But the activity of succinate dehydrogenase was similar in both groups. This result may be due to encoding of some cytochrome C oxidase subunits by mitochondrial genome whereas total subunits of succinate dehydrogenase are encoded by nuclear genome. Similar to our results, Li et al. showed that impaired mtDNA could lead to decrease in cytochrome C oxidase activity, but succinate dehydrogenase activity did not change (64).

Our molecular analysis demonstrated that TFAM gene expression in vitrified MII oocytes was lower than non-vitrified group. This result may be due to the key role of TFAM in stability of mitochondrial genome (mtDNA). Kukat et al. reported that, there are 1000 TFAM molecules for one mtDNA in each nucleoid. TFAM molecules have important regulatory role in biogenesis and stability of mtDNA (65, 66). Thus, decrease in mtDNA copy number may be due to decrease in TFAM gene expression.

## Conclusion

This study showed that vitrification of mouse MII oocytes caused some alterations in the oocytes mitochondria. Reducing in the mtDNA copy number, mitochondrial cytochrome C oxidase activity and increase in ROS level could affect the subsequent embryo development. The improvement of oocyte vitrification method is recommended.
